# Attributes of Expert Anticipation Should Inform the Design of Virtual Reality Simulators to Accelerate Learning and Transfer of Skill

**DOI:** 10.1007/s40279-022-01735-7

**Published:** 2022-07-26

**Authors:** Sean Müller, Evan Dekker, Khaya Morris-Binelli, Benjamin Piggott, Gerard Hoyne, Wayne Christensen, Peter Fadde, Leonard Zaichkowsky, John Brenton, David Z. Hambrick

**Affiliations:** 1 Research Centre for Smart Analytics, Federation University Australia, University Drive, Mt. Helen, Ballarat, VIC 3350 Australia; 2 Academic Services and Support Directorate, Federation University Australia, University Drive, Mt. Helen, Ballarat, VIC 3350 Australia; 3 School of Health Sciences and Physiotherapy, The University of Notre Dame Australia, Perth, Western Australia; 4 School of Philosophy, University of Barcelona, Barcelona, Spain; 5 School of Education, Southern Illinois University, Carbondale, IL USA; 6 Performance Consultant, Fort Myers, Florida USA; 7 Performance Consultant, Perth, Western Australia; 8 Department of Psychology, Michigan State University, East Lansing, MI USA

## Abstract

Expert sport performers cope with a multitude of visual information to achieve precise skill goals under time stress and pressure. For example, a major league baseball or cricket batter must read opponent variations in actions and ball flight paths to strike the ball in less than a second. Crowded playing schedules and training load restrictions to minimise injury have limited opportunity for field-based practice in sports. As a result, many sports organisations are exploring the use of virtual reality (VR) simulators. Whilst VR synthetic experiences can allow greater control of visual stimuli, immersion to create presence in an environment, and interaction with stimuli, compared to traditional video simulation, the underpinning mechanisms of how experts use visual information for anticipation have not been properly incorporated into its content design. In themes, this opinion article briefly explains the mechanisms underpinning expert visual anticipation, as well as its learning and transfer, with a view that this knowledge can better inform VR simulator content design. In each theme, examples are discussed for improved content design of VR simulators taking into consideration its advantages and limitations relative to video simulation techniques. Whilst sport is used as the exemplar, the points discussed have implications for skill learning in other domains, such as military and law enforcement. It is hoped that our paper will stimulate improved content design of VR simulators for future research and skill enhancement across several domains.

## Key Points

**Table:** 

This opinion piece discusses key mechanisms that underpin expert visual anticipation in sport and how this knowledge can inform the design of virtual reality simulators to better assess and train anticipation skill.
If virtual reality simulator design does not incorporate the mechanisms of expert visual anticipation, these systems could impede or regress athlete performance.

## Introduction

Expert sport performers are consistently able to execute precise skills under high time and pressure constraints but give the impression of having *all the time in the world* [1]. While it is easy to attribute these extraordinary skills to inborn talent and/or accumulated experience, the question posed by coaches, athletes, and sports scientists is whether acquisition of these skills can be accelerated through training. In particular, can simulator-based training that takes advantage of ever more advanced and affordable virtual reality technologies offer a path for accelerated expertise? An intriguing article was published in *Nature Outlook* [2] on this topic, which presented a science journalist’s interpretation of research on how expert athletes use visual information to execute their skills and simulators that have been designed to potentially accelerate athlete learning. The article discussed several themes including information sources, perception–action coupling, expert versus novice differences, and virtual-reality (VR) simulator content design. Knowledge from such themes is typically used to design content of simulators to facilitate learning and transfer of skill.

We define VR as cave automatic virtual environment (CAVE) or head-mounted displays (HMD), such as Oculus Quest, that fully immerse the user to create the presence of being in a different environment to the current location [3]. These VR systems focus upon the key principles of presence, interactivity, and immersion using synthetic or video-based experiences [3]. It has been reported that presence (defined as the experience of being in a simulated context) and interactivity (defined as the degree to which the user can influence the virtual environment) contribute to immersion (defined as being absorbed in an activity) within VR [3]. Cave automatic virtual environment VR and HMDs can include synthetic stereoscopic stimuli that present graphical representations of the environment and individuals, with body positional tracking of motor responses [4]. Collectively, synthetic VR systems offer a higher degree of control over presented stimuli and interaction with motor responses, compared with video-based VR experiences, in order to maximise immersion. Head-mounted systems can also present a simulated environment and individuals as a video-based experience [4]. Such systems offer lower control over presented stimuli than synthetic systems and no interaction with motor responses, but immersion can still be maintained [4]. Our view in terms of future use of VR for research or skill development is that a crucial starting point is the design of visual-perceptual information used for anticipation performance and learning.

The beginning of Drew's [2] article states that experts pick-up visual information from contextual, postural (kinematic), and ball flight cues to guide fast actions. Later in the article, however, it is mentioned that more recent VR simulator design in sport has focused upon placing the performer within a specific environment such as a tennis arena and implementing overt perception–action coupling. This focus on physical fidelity (i.e., realism or presence of the simulation) is concerning because it can overlook psychological fidelity that refers to the perceptual, cognitive, and motor mechanism underpinning expert skill [5]. As a result, VR that looks and feels real, but that does not adequately model the mechanisms of performance and learning, may be inefficient or even potentially counterproductive [5]. In sports, there are a range of factors that need to be taken into consideration regarding how experts use visual information to guide action. Inadequate attention to these factors in the design of VR simulators can cause athletes to attend to later visual information, such as object flight, that can delay their actions in high time-constrained sports such as baseball, tennis, cricket, or field hockey [1]. Therefore, it is important to reflect upon the themes in Drew's [2] paper relative to the broader expert anticipation literature to ensure that VR simulators are better designed to facilitate, rather than regress, skill learning and transfer.

The purpose of this brief opinion paper is to discuss what is currently known about expert pick-up of visual information for anticipation and how it can be trained, with a view that this knowledge can better inform VR simulator content design. Anticipation is defined as the capability to use visual (e.g., opponent kinematics) and non-visual (e.g., game score) information to predict what will happen, before or early in an opponent’s action, and make a correct decision using verbal, button press, or motor responses [6]. This paper is focused upon the critical skill of anticipation because in striking or open-field play sports, performers need to make or inhibit fast actions with minimal error tolerance under pressure [7]. Our paper is structured into themes that allow discussion of the mechanism underpinning pick-up of visual information in order to perform and improve skill. The themes discussed include information sources, point-light and blurred displays, individual differences, perception–action coupling, early and late visual information, as well as learning and transfer. In each theme, considerations are outlined for improved VR simulator content design and integration with well-established video-based simulation, with a view that both types of simulators can be more easily incorporated for future research and within standard practice schedules for skill development. The following section discusses factors relating to visual anticipatory performance and learning in sport.

## Factors Contributing to Anticipation Performance and Learning in Sport

### Information Sources

Video or field-based temporal occlusion, where the time course of visual information is controlled through a black video frame or glasses that switch from clear to opaque, has been predominantly used to understand anticipation in sport [6]. Alternatively, in video-based spatial occlusion, selected segments of the simulated scene such as an opponent’s tennis racquet are masked (occluded) in order to determine the sources of information used for anticipation in sport [6]. Through video-based temporal and spatial occlusion, it is well established that expert performers are superior to novices in their capability to use advance (pre-object flight) postural (kinematic) cues in the movements of an opponent in order to anticipate [8]. Advance information is used for initial body positioning to strike an object, save a shot on goal, or change direction when defending [9–11]. In the past decade, more fine-grained evidence has emerged concerning how experts use advance information. Studies have reported that experts are superior to lesser skilled players in their capability to pick-up both contextual and kinematic information to anticipate [12, 13]. Contextual information can be visual or non-visual such as information from memory of field-placings in cricket batting or opponent action tendencies in soccer, respectively [10, 14]. It has been reported that experts have a superior capability for integrated pick-up of contextual and kinematic information, and this is magnified dependent upon whether there is congruence (i.e., both sources of information suggest the same outcome), rather than incongruence (i.e., one source of information suggests an outcome, while the other does not), between these sources [14]. The capability to integrate contextual and kinematic information is a crucial mechanism to guide initial body positioning for achievement of the skill goal [1, 15]. There is evidence, however, that some experts rely solely upon contextual information even if it is congruent with kinematic information [16]. This can be a major concern as overreliance on contextual information such as opponent action tendencies can make the performer susceptible to being deceived by the opponent’s kinematics [10]. For example, in baseball, when a pitcher is behind the count, a batter may predict a fastball will be thrown, so the batter may place less emphasis on kinematic cues, but the pitcher may throw a curveball or change-up. Therefore, the capability to switch between contextual and kinematic information to anticipate is critical because opponents can intentionally or unintentionally alter kinematics relative to their skill goal [17].

#### Options to Better Simulate Advance Cues

As observed by Drew [2], some existing VR simulators do not present advance kinematic cues with temporal occlusion to challenge users’ early visual information pick-up. This is concerning as experts use postural (kinematic) information to gain time for initial body positioning in a range of sports skills including cricket and baseball batting, returns of serve in tennis, and goalkeeping in soccer and field hockey, to name a few [1, 17]. A possible reason that kinematic cues are not properly simulated in VR is due to the difficulty associated with accurately modelling and animating in a synthetic experience the intricate details of the fingers, hand, and arm connections such as of a baseball pitcher or cricket bowler. To overcome this limitation, 360° stereoscopic video footage of a pitcher could be captured and presented with temporal or spatial occlusion in an HMD. In addition, contextual information such as the pitch count in baseball could be presented to be congruent or incongruent with kinematic and ball flight information. The user can be required to make a verbal, button press or simulated motor response to advance cues (see perception–action coupling section). These improved content design features of VR attempt to maintain presence and immersion, but importantly exposure to crucial advance cue information sources (psychological fidelity). Accordingly, the capability of the user to pick-up crucial advance cues with control over their presentation through temporal occlusion will help determine in a controlled manner, whether performers can use these advance sources of information that are relevant to body positioning [17]. Thereafter, temporal and spatial occlusion can be applied in combination during object flight to challenge pick-up of this information for object interception [17]. Therefore, including these features into VR content design would take into consideration the mechanism of expert anticipation that maps the pick-up of temporally evolving sources of visual information to components of the performer’s action response.

### Point-Light and Blurred Displays

Video simulation of an opponent(s) movements have been displayed as points-of-light placed on anatomical landmarks with a black background or where the display has been blurred [9, 18]. The purpose of these methods is to remove featural information such as colour, contour, or shape, but preserve configural (relation) information [19]. Several video-based studies have reported that experts, but not lesser skilled players, use the minimal (configural) information from kinematics presented in point-light and blurred displays to anticipate [18, 20, 21]. This low-resolution visual information is suggested to be related to the peripheral visual field and dorsal visual stream that processes visual information at a fast sub-conscious level [22]. In a related manner, pioneering work by Abernethy and Russell [8] using video temporal and spatial occlusion, reported that whilst expert badminton players verbalised use of racquet-shuttle contact information to predict shuttle landing location, they actually relied upon information from the arm holding the racquet to strike the shuttle. Therefore, it appears that there is an intricate interplay between pick-up of contextual information (mentioned in previous section) at a more conscious level [e.g., 14], and pick-up of kinematic information at a sub-conscious level [8] for visual anticipation.

#### Options for Simulated Display Resolution

Drew [2] mentions the “low-resolution” avatar in a tennis VR simulator as a technological limitation. Indeed, the focus of several VR simulators is to present high-resolution displays such as the setting of an actual sports arena that create the performer’s presence relative to the real-world context [4, 23]. Whilst presence is important for user engagement in VR simulators [3], it is not crucial for anticipation performance and learning in sport. As mentioned above, it is important to note that experts rely upon low-resolution configural, rather than high-resolution featural, information, to anticipate. Accordingly, studies have compared visual-perception of handball thrower kinematics using point-light, wire-frame, no texture, and synthetic experiences in VR, with no differences reported in performance across the different experiences [24]. Therefore, VR simulators could be designed to incorporate a combination of high-resolution displays, as well as point-light and blurred displays to ensure that sub-conscious visual processes are targeted for the pick-up of advance and object flight information [24, 25]. Such manipulations applied to video simulation have shown improvements to anticipation in expert athletes [26] and novice [27] performers in sport, which can be manipulated in synthetic or video-based VR experiences. A combination of high- and low-resolution displays will ensure that presence, immersion, and interactivity are intricately balanced with psychological fidelity that targets the fast sub-conscious processes of anticipation.

### Individual Differences

Whilst the studies discussed above have been useful, they have focused upon *group*-based comparisons, rather than individual differences within expert samples [28]. Drew [2] addresses this point effectively by discussing an individual differences study by Land and McLeod [29] that reported a professional cricket batsman made earlier predictive saccades to future ball landing positions, compared to amateurs, in order to strike balls. Predictive saccades position the eyes ahead of the ball to cope with time constraints for interception and further confirms that anticipatory control occurs in field-settings. More recently, using video temporal occlusion, it has been reported that there are differences between expert field hockey goalkeepers (of international and national level) in their capability to integrate congruent contextual and kinematic information to anticipate a drag-flick on goal [15]. This indicates that the mechanism to switch between contextual and kinematic information may not be well developed in all expert athletes. Accordingly, it is important to consider the individualised nature of anticipation skill, which can be dependent upon experience, expertise, and/or consistent exposure to practice environments that challenge the integrated pick-up of contextual and kinematic information [30]. Moreover, some athletes may have superior capability to pick-up visual information, but inferior motor response timing or vice versa. Therefore, an important goal of research is to identify why inter- and intra-individual differences exist, whilst for skill development it is important to target relative deficiency for training.

#### Level of Analysis in Simulated Contexts

Where possible, design of VR should incorporate nested individual comparisons for research and application in skill development. Due to the capability for body positional tracking in synthetic VR experiences, it is possible for anticipation outcomes such as response timing and response accuracy to be measured on a continuous scale. Whereas for video-based VR experiences, anticipation outcomes for response timing can still be measured on a continuous scale, with response accuracy on a dichotomous scale. This level of analysis in VR is valuable for three reasons. First, as competition skill level increases there is a smaller sample of genuine experts and it is challenging to recruit large samples for studies that include field-based assessments [31]. Adequate statistical power can be attained through sufficient individual experimental trials, which will allow investigation of individualised integration of contextual and kinematic information for anticipation [15]. Second, individual visual information pick-up and motor capability can be probed through combined temporal occlusion and response accuracy timing, respectively. This is useful to determine whether anticipation training should target visual-perceptual and/or motor responses for body positioning and interceptive phases of a skill. Third, practitioners including coaches, scouts, and sports scientists want to know the performance level of individual athletes for talent identification and/or skill development [28, 30]. Therefore, an individual differences approach can cater to research and skill development needs by targeting the underpinning mechanism of anticipation.

### Perception–Action Coupling

There continues to be debate regarding the contribution of perception and/or overt action coupling to anticipation skill [32]. The literature is inconclusive in terms of whether visual-perception to overt action-coupled responses facilitate superior anticipation over perception-only responses. Some studies have reported superior anticipation based upon pick-up of advance kinematic information with an overt action response compared to a verbal (perception-only) response [33], whilst others have reported no advantage of perception to overt action coupling relative to perception-only responses [32, 34]. Moreover, studies have indicated that experts, but not less-skilled players, can predict above the guessing level based upon pick-up of kinematic information using a verbal response [35 see Experiment 1], and timing of visual information pick-up is consistent across verbal and overt action responses modes [36, 37 see introduction of latter]. Three important factors need to be considered to clarify how perception and/or overt action influences anticipation. First, the presentation of sports-specific kinematic information and its use relative to expertise is a key component of anticipatory skill [38]. Second, several neurophysiological studies have reported that sensory and motor regions of the brain are finely tuned, and in turn ‘coupled’, to sports-specific perceptual information when the performer is stationary [39, 40]. In a field setting of the motor skill, this neural coupling is, of course, necessary to prepare the body to initiate or inhibit action in a timely manner and for on-going action control [39]. Third, evidence indicates that the motor system is engaged (prior to action) to simulate the observed action in order to anticipate its outcome [41]. Therefore, perceptual (e.g., verbal) and/or motor responses are suitable for assessment and training anticipation skill [37 p2].

#### Flexibility in the Use of Perceptual and Motor Responses

Based upon the above, VR simulator content design can incorporate verbal, button press, and/or motor responses to sports-specific perceptual information. This may be necessary for two reasons. First, a verbal or button press response will allow easy use of commercial video-based VR experiences that can be cost-efficient and re-create sports-specific information, but are not sufficiently advanced like more expensive synthetic experiences to measure perceptual-motor positional or accuracy responses at high sampling frequencies [42, 43]. Second, task difficulty and challenge may require progression from perception-only to perception–action responses relative to the individual and skill level [44]. Synthetic VR experiences, however, provide greater control over visual stimuli with opportunity for tighter coupling of motor responses [42]. This is invaluable to several sports such as baseball or cricket, where batters can be assessed and trained against a variety of simulated opponents. It is noteworthy, however, that in other domains such as military and law enforcement, VR simulators indeed incorporate a combination of verbal, button press, or simplified motor responses [4]. The crucial design feature is to ensure that key visual-perceptual information is displayed (as mentioned earlier) to ensure that presence and immersion, as well as interaction (where relevant) are maintained so that a variety of instructional methods can be used to draw sub-conscious attention to these information sources for improved anticipation (see below).

### Early and Late Visual Information for Action

The previously discussed content has considered the pick-up of early visual information for anticipation. Some field-based studies have also used spatial occlusion, which involves masked advance or object flight cues, with performance decrements relative to the unoccluded control condition indicating the importance of the masked (occluded) scene for anticipation [45]. Using spatial occlusion, studies have reported that experts require opponent kinematic information to make initial action responses to position their body [45]. Related field-based temporal occlusion studies have reported that experts are superior to lesser skilled players in their capability to use earlier occurring contextual [46] and kinematic [11] information to position their body. Together, these studies have confirmed that anticipatory control of gross body positioning occurs in the field-setting of the sport. Field-based temporal and spatial occlusion studies have also reported that experts use later occurring object flight information to fine-tune action during the interception phase [47]. This finding indicates that experts also use a prospective (pick-up of ongoing information) control strategy when time permits to adjust later occurring motor responses. Collectively, the findings of these field-based studies are in line with expert use of advance and object flight information to make object depth/direction (related to body positioning) and location (fine-tuning action) predictions in video-based temporal occlusion tasks, respectively [14, 38]. Therefore, it appears that expert performance involves an intricate blend of predictive and prospective control strategies [1]. Nonetheless, it is important to consider that in high time-constrained skills, if predictive control is not well developed, it is unlikely that there will be time for fine-tuning of action through prospective control [48].

#### Simulation of Early and Later Visual Information

To target both predictive and prospective control, VR simulators can be designed where temporal occlusion is applied immediately after presentation of advances cues, but also during object flight. This would ensure that performers must attend to both early visual information that maps onto gross body positioning and later visual information that maps onto fine-tuning of action [1]. Synthetic VR simulators offer a range of options where temporal and spatial occlusion can be applied throughout the course of an opponent’s action and object flight. For example, spatial occlusion can be applied to a segment of the pitcher’s body (early cues), with temporal occlusion applied to sections of only the ball’s flight (late information), whilst the surrounding synthetic experience is maintained [49]. This can maintain immersion, interaction, and presence within VR, whilst selectively manipulating early and late information used for anticipation. Currently, VR simulators are not consistently designed to probe this early and late visual-motor mapping [2], which can draw the user’s focus towards late object flight information to inform action that is a characteristic of lower skilled performers.

### Learning and Transfer

A variety of instructional approaches have been used to improve anticipation skill. These include; temporal occlusion with an unoccluded replay [50, 51], verbal cueing to key kinematic cues [52], desensitisation through training under anxiety [53], use of point-light and blurred displays with an unoccluded replay [26, 27], above real-time training [54], visual-perceptual and motor practice of the observed action [26, 55], and imagery [56]. These studies have predominantly reported improvements in the pick-up of kinematic information for anticipation, which would allow more accurate and timely predictive control of body positioning [1, 11]. Other studies have reported that anticipation can be improved and transfers to field-settings using perception and/or overt action response modes [57, 58]. Some studies that trained generic or perceptual visual skills have not reported transfer to field settings [e.g., 54, 59]. Lack of transfer for generic visual skill training is due to the perceptual information not involving postural cues, whilst for the perceptual training study, this may have been limited by participant sample size. Related to the earlier perception–action coupling section, the findings of anticipation training studies mentioned above indicates that improved pick-up of kinematic information can occur whether the performer is static or in motion. This is likely (as mentioned earlier) because the neuromotor system of experts is better attending to key visual-perceptual information than less-skilled players. Like performance studies discussed in previous sections, more recently, improvement in the pick-up of integrated contextual and kinematic information with transfer to field-settings has been reported to be individualised in expert samples [15]. This can be due to individualised capabilities to integrate perceptual information, which might be due to consistent lack of or reduced exposure to these cues in practice settings [30]. Collectively, the reported range of instructional approaches for improved pick-up of advance cues for anticipation presents a variety of techniques that can be also used in VR to accelerate learning and transfer.

#### Approaches for Improving Performance

The points discussed in previous sections need to be taken into consideration when designing VR simulators for learning and transfer. Whilst considerable work still needs to be done to determine whether VR training improves performance and facilitates transfer, there is no reason to suggest that the instructional approaches validated through video simulation will not be at least of equal value in the VR medium. In terms of synthetic VR simulators, there is greater control over the manipulation of stimuli relative to action responses and selective presentation of correct or false feedback, than in video-based VR. Whether these benefits indeed lead to greater learning and transfer of anticipation than video-based VR and video-based simulation is yet to be established. Further, there are current challenges in creating accurate synthetic models of opponent kinematic cues (see Sect. 2.1). Collectively, this indicates that sporting organisations and practitioners should carefully consider whether the time and funds required to create synthetic VR simulators that have adequate psychological fidelity is the most viable option to assess and train anticipation skill.

Whether video-based VR compared to video-based simulation leads to superior learning and transfer of anticipation also remains to be confirmed. For example, a recent study by Fortes et al. [60] compared video-based simulation and video-based VR simulator training for decision-making in soccer. In this study, a single monoscopic GoPro video camera was used to capture open-field-play scenario footage, rather than use of a stereoscopic camera that is a key feature of VR [61]. Stereoscopic vision has been reported to maximise cues for depth perception, as well as performers’ sense of immersion and presence within the VR environment [61]. Further, monoscopic cameras provide a limited field of view compared to available 360° stereoscopic cameras [4]. Fortes et al. [60] reported both simulators improved decision-making (choice response) accuracy, but VR was superior (moderate effect size). If 360° stereoscopic footage was used in their study, this may have resulted in greater benefits particularly for open field sports such as soccer or Australian football where rotation of the head is necessary to acquire information down the field and in close proximity to opponents. It is also worth noting that the greater improvements to decision-making in a field-based setting by the video VR compared to the video-based simulation training group cannot necessarily be attributed to the VR training allowing greater overt perception–action coupling. This is because the participants’ action responses in video VR have no direct influence on the perceptual stimuli presented (interactivity). It could be possible, however, that greater immersion in video VR, along with simulated field play scenarios, leads to improved participant engagement during training, and this in turn leads to greater learning and transfer [42].

Due to the reasons mentioned above, careful consideration needs to be given as to whether and how video and VR simulation should be compared and used. It may well be that both could be used within their scope to improve the anticipatory skill of athletes in different sports, with different levels of development or competition, and with different deficiencies to address. For example, to improve athletes’ capabilities to pick up contextual and kinematic advance cues, video-based simulation or video-based VR could be used, while to improve athletes’ pick-up of early object flight to fine-tune motor responses, synthetic VR could be used. Alternatively, in a single VR simulator, contextual and kinematic advance cues could be presented via video simulation, whilst object flight is synthetically simulated [62].

## Conclusion

VR simulators are widely used for assessment and training of skill in sport and other domains [2, 4]. Nevertheless, the validity of VR simulators in the sport domain has not been properly established compared to video simulation. The goal of this paper was to raise awareness that current content design of VR simulation in sport has not taken into complete consideration the existing body of literature on expert visual anticipation. Existing design features can have significant consequences for the athlete such that it may draw their attention to later occurring visual information, which may in turn, make their responses later and rushed. The flow on effect is that practitioners who use inadequately validated and designed VR could direct their athletes down a path of detraining or less expert-like motor behaviour. Through more appropriate design of VR simulators based upon existing expert anticipation knowledge, these concerns can be rectified at least in the short-term. A systematic series of experimental investigations are required to establish that the expertise differences and mechanisms observed through video simulation and field-based techniques discussed herein are consistently observed in VR simulators. Beyond confirmatory research on VR, a design-based research agenda should also target specific VR features as independent variables (e.g., video vs. synthetic experiences). Failure to proceed with caution could mean that athletes and performers in other domains who train with VR are, at best, paying for unnecessary simulator physical fidelity and, at worst, being ill prepared to execute their skills to maximal capability.
