# Correction to: Attributes of Expert Anticipation Should Inform the Design of Virtual Reality Simulators to Accelerate Learning and Transfer of Skill

**DOI:** 10.1007/s40279-022-01745-5

**Published:** 2022-08-02

**Authors:** Sean Müller, Evan Dekker, Khaya Morris-Binelli, Benjamin Piggott, Gerard Hoyne, Wayne Christensen, Peter Fadde, Leonard Zaichkowsky, John Brenton, David Z. Hambrick

**Affiliations:** 1 Research Centre for Smart Analytics, Federation University Australia, University Drive, Mt. Helen, Ballarat, VIC 3350 Australia; 2 Academic Services and Support Directorate, Federation University, University Drive, Mt. Helen, Ballarat, VIC 3350 Australia; 3 School of Health Sciences and Physiotherapy, The University of Notre Dame Australia, Perth, Western Australia; 4 School of Philosophy, University of Barcelona, Barcelona, Spain; 5 School of Education, Southern Illinois University, Carbondale, IL USA; 6 Performance Consultant, Fort Myers, Florida USA; 7 Performance Consultant, Perth, Western Australia; 8 Department of Psychology, Michigan State University, East Lansing, MI USA

## Correction to: Sports Medicine 10.1007/s40279-022-01735-7

**Page 1:** The affiliation for Evan Dekker, which previously read:

^2^Academic Services and Support Directorate, University Drive, Mt. Helen, Ballarat, VIC 3350, Australia

has now been updated to read:

^2^Academic Services and Support Directorate, Federation University, University Drive, Mt. Helen, Ballarat, VIC 3350, Australia

The original article has been corrected.

